# Latent profile analysis of self-neglect and associated factors among rural older adults with chronic diseases: a cross-sectional study

**DOI:** 10.3389/fpubh.2026.1738418

**Published:** 2026-01-28

**Authors:** Zihan Yi, Chengchuan Chen, Zikejimu Sun, Chaoxiang You, Mei Ju, Na Zhou

**Affiliations:** 1 Department of Nursing, People’s Hospital of Deyang City, Deyang, China; 2 School of Nursing, Southwest Medical University, Luzhou, Sichuan Province, China; 3 Department of Anesthesiology, The Affiliated Hospital of Southwest Medical University, Luzhou, Sichuan Province, China; 4 Department of Pediatrics, People’s Hospital of Deyang City, Deyang, China; 5 Department of Gastroenterology, People’s Hospital of Deyang City, Deyang, China; 6 Department of Cardiology, The First Affiliated Hospital, Chengdu Medical College, Chengdu, Sichuan Province, China

**Keywords:** chronic diseases, latent profiles, older adults, rural, self-neglect

## Abstract

**Objective:**

This study aimed to identify heterogeneous profiles of self-neglect (ESN) and their associated factors among rural Chinese older adults with chronic diseases.

**Methods:**

A cross-sectional survey was conducted among 719 rural older adults with chronic diseases in Sichuan, China, from January to June 2020. The questionnaire included sociodemographic and health-related characteristics, as well as the Three-Item UCLA Loneliness Scale (UCLALS-3), the Social Support Rating Scale (SSRS), the Scale of Older Adults Self-Neglect (SESN), the Five-Item Geriatric Depression Scale (GDS-5), and the Short Portable Mental Status Questionnaire (SPMSQ). Latent profile analysis (LPA) was conducted to identify distinct patterns of patterns of self-neglect among older adults (ESN).

**Results:**

Four profiles were identified: low-level neglect (35.0%), selective mild neglect (37.7%), moderate neglect (14.7%), and severe neglect (12.5%). Compared with the low-level neglect group, selective mild neglect was more common among participants with poorer economic status, poor sleep quality, and alcohol consumption. The moderate neglect profile was associated with older age, lack of regular physical examinations, smoking, pain, cognitive impairment, and lower social support. Severe neglect was marked by the absence of grandchild caregiving, higher loneliness, smoking, and depression. Pairwise comparisons indicated stage-dependent patterns, with reversed associations for social support (protective in moderate neglect but a risk marker in severe neglect) and pain (a risk factor in moderate neglect, whereas its absence indicated higher risk in severe neglect).

**Conclusion:**

ESN among older adults with chronic diseases in rural China is heterogeneous and comprises distinct latent profiles with stage-dependent risk factors. For selective mild neglect, interventions should emphasize economic and lifestyle support. For moderate neglect, priorities include routine monitoring, regular physical examinations, and health literacy promotion. For severe neglect, intensive psychosocial interventions should address depression and loneliness and promote alternative engagement in family roles, particularly among older adults who do not provide grandchild caregiving. Integrating these profile-specific strategies into rural primary care may help reduce self-neglect and improve health outcomes in this vulnerable population.

## 1 Introduction

Global population aging is accelerating as life expectancy increases and fertility rates decline. The number of people aged 60 years and older is projected to more than double, from 901 million in 2015 to over 2.1 billion by 2050 (1). In China, the population aged 65 years and older reached 220.23 million by the end of 2024, accounting for 15.6% of the total population (2). This demographic shift is increasing pressure on health care systems, particularly in rural areas where aging is more pronounced and health care infrastructure is limited (3, 4). Between 1990 and 2020, the proportion of rural residents aged 65 years and older tripled, reaching about 18% by 2020 (5). Together, these figures underscore the rapid pace of aging in rural China.

Population aging is accompanied by a rising burden of chronic disease among older adults (6). In rural China, the prevalence of chronic conditions was high, reaching 60% between 2008 and 2018 (7). Rural communities also face constrained access to care, inadequate infrastructure, and reduced family support related to migration (8). In combination, these conditions can complicate health management in later life (9, 10) and increase susceptibility to self-neglect, a critical health problem that is often overlooked.

Self-neglect among older adults (ESN) refers to failures to meet basic needs, including nutrition, hygiene, and safety, due to an inability or unwillingness (10). A recent systematic review estimated the prevalence of ESN among older adults at approximately 28% (11). As a global public health concern, ESN threatens the health and safety of older adults and hinders progress in health promotion (12). Prior studies have linked higher ESN levels to lower quality of life (13), poor nutritional status (14), reduced medication adherence (15), and higher hospitalization rates (16). ESN can undermine chronic disease management, and chronic diseases may further increase vulnerability to neglect (17). In rural settings, effective management often requires greater self-efficacy and proactive health behaviors. Compared with urban older adults, rural older adults with chronic conditions carry a greater self-management burden, including complex medication regimens, dietary restrictions, and long-term symptom monitoring (18). These challenges support the need for targeted public health interventions and community-based strategies for this vulnerable population.

Most ESN studies have examined community-dwelling or generally healthy older adults and selected subgroups, including rural older adults without major health conditions and those living alone (17, 19, 20). When chronic disease has been included, research has typically focused on single conditions, such as hypertension or diabetes, and has prioritized disease self-management over self-neglect (21, 22). In addition, ESN has often been modeled as a continuous outcome in regression-based analyses (23, 24). Although informative, these approaches can obscure heterogeneity in how self-neglect presents, which is particularly salient in rural contexts where limited access to care, reduced social support, and economic hardship shape daily functioning. Overall, the ESN literature can be summarized into three strands: community-based studies in generally healthy older adults that estimate prevalence and correlates; service-based studies using Adult Protective Services (APS) or clinical records that capture more severe cases but may not generalize to rural community populations; and disease-specific studies that emphasize chronic disease self-management rather than ESN as a multidimensional syndrome. In China, empirical evidence on ESN is still emerging, and studies focusing on rural older adults with chronic diseases remain scarce.

Latent Profile Analysis (LPA) can identify subgroups defined by patterns of ESN rather than assuming a homogeneous presentation. Despite its relevance, LPA has rarely been used to classify ESN subtypes. Burnett et al. (25) identified four patterns of self-neglect among older adults reported to APS, but that work relied on social service records from a general older population, which limits applicability to rural older adults with chronic diseases. Building on prior work, this study applies LPA to characterize heterogeneous ESN patterns among rural older adults with chronic diseases in China and examines profile membership in relation to sociodemographic, psychosocial, and behavioral factors relevant to rural chronic disease management. The findings may inform rural health policy and support the development of profile-informed public health strategies and care models for this vulnerable population.

## 2 Methods

### 2.1 Study design

This cross-sectional study was conducted among rural older adults in Sichuan Province, China, using a multi-stage stratified convenience sampling approach. Data were collected from January to June 2020. Stratified convenience sampling was used to facilitate fieldwork while capturing socioeconomic diversity within the rural population. Stratification was based on county and per capita gross domestic product (GDP), as reported in the 2018 Sichuan Statistical Yearbook, to represent different socioeconomic subgroups. Sichuan was selected because it has a large, diverse rural population with substantial variation in geography, socioeconomic conditions, and health care access. Variation in local infrastructure and health care services also supports its suitability for examining factors related to self-neglect among older adults.

### 2.2 Participants

Participants were eligible if they (1) were aged 60 years or older, (2) had a clinical diagnosis of at least one chronic condition, including diabetes, hypertension, coronary heart disease, stroke, chronic obstructive pulmonary disease (COPD), or cancer, and (3) provided written informed consent after a full explanation of the study. Exclusion criteria were (1) withdrawal before study completion, (2) terminal illness, or (3) severe communication disorders that precluded participation.

### 2.3 Sample size

According to Nylund-Gibson et al. (26), at least 300 participants are required to ensure stable parameter estimation in LPA. On the basis of this recommendation and the study design, the required sample size was calculated using the following formula:


$$n=\frac{Z_{1-\alpha /2}^{2}\times p\;(1-p)}{d^{2}}$$

In this calculation, Z = 1.96 for a 95% confidence interval (CI), the expected prevalence (*p*) was 27% (27), and the allowable margin of error (*d*) was 0.035. The minimum required sample size was 619. Allowing for an anticipated 10% non-response, the target sample size was increased to 688 participants.

### 2.4 Measures

#### 2.4.1 General information questionnaire

A structured questionnaire was used to collect participants’ basic characteristics, including age, sex, living arrangement (alone vs. not alone), widowed status, self-rated economic status (good/fair/poor), grandchild caregiving (yes/no), frequency of communication with children, perceived loneliness, self-rated personality (introverted/neutral/extroverted), physical examination status, mobile phone use (smartphone/basic phone/non-user), sedentary behavior, sleep quality (good/fair/poor), self-rated health, smoking, alcohol consumption, pain, and number of chronic conditions. Frequency of communication with children was assessed by the question “How often do you communicate (in person or by phone) with your children?” with responses categorized as >3 times a week, 1–3 times a week, once a month, or less than once per month (28). Sedentary behavior was defined as sitting or reclining for more than 4 h per day, excluding sleep time, based on thresholds associated with adverse health outcomes in older adults (29, 30).

#### 2.4.2 Three-item UCLA loneliness scale (UCLALS-3)

Perceived loneliness was measured with the UCLALS-3 developed by Hughes et al. (31). The scale includes three items assessing loneliness during the past week. Responses are recorded on a 3-point scale (1 = hardly ever, 2 = some of the time, 3 = often). Total scores range from 3 to 9, with higher scores indicating greater loneliness. In this study, a cutoff score of 6 or higher was used to indicate elevated loneliness (32). The Cronbach’s alpha in this study was 0.824.

#### 2.4.3 Social support rating scale (SSRS)

Social support was assessed using the SSRS, developed by Xiao (33). This scale evaluates three dimensions of social support: objective support, subjective support, and support utilization. The SSRS consists of 10 items, with higher scores indicating greater levels of support. Objective support refers to the tangible support available, such as the number of people providing assistance; subjective support measures the perceived adequacy of support; and support utilization assesses the actual use of available support. The total score, which sums the scores from all three dimensions, provides an overall measure of social support. Previous studies reported good internal consistency (Cronbach’s alpha, 0.89 to 0.94) and test–retest reliability of 0.923 (33). In the current study, Cronbach’s alpha was 0.707.

#### 2.4.4 Scale of older adults self-neglect (SESN)

ESN was assessed with the SESN developed by Zhao (13) to evaluate self-neglect behaviors in rural Chinese populations. The SESN contains 14 items across five dimensions: (1) medical health and care (Items 1 to 3), assessing medical compliance and health maintenance behaviors, including seeking medical care, following medication instructions, and maintaining appropriate diet and nutrition; (2) environmental sanitation and personal hygiene (Items 4 to 6), assessing home cleanliness, personal hygiene, and household maintenance; (3) mental health (Items 7 to 9), assessing emotional well-being and self-perceived psychological regulation; (4) safety (Items 10 to 12), assessing awareness and preventive behaviors related to fire hazards, falls, and household safety; and (5) social communication (Items 13 to 14), assessing interpersonal contact and willingness to seek help during illness or emergencies. Items are rated on a 4-point Likert scale from 0 (never) to 3 (always). Total scores range from 0 to 42, with higher scores indicating more severe self-neglect. The Cronbach’s alpha in this study was 0.752.

The SESN is suitable for LPA because its five dimensions capture distinct yet related aspects of self-neglect. Its item scoring (0 to 3) yields a broad total score range and supports identification of subgroups. Because the scale emphasizes observable behaviors, including hygiene, safety practices, and social engagement, it aligns with the person-centered framework of LPA. For reproducibility and more detailed profiling, the 14 individual SESN items were used as indicators in the LPA rather than aggregated dimension scores.

#### 2.4.5 The five-item geriatric depression scale (GDS-5)

Depressive symptoms were assessed using the GDS-5, developed by Hoyl et al. (34). The scale includes five items and serves as a brief screening tool for depressive symptoms in older adults. Each item is scored 0 (absence) or 1 (presence), yielding a total score from 0 to 5. A score of 2 or higher indicates depression. The Cronbach’s alpha in this study was 0.770.

#### 2.4.6 Short portable mental status questionnaire (SPMSQ)

Cognitive status was assessed with the 10-item SPMSQ developed by Pfeiffer et al. (35). The SPMSQ evaluates memory, orientation, and general cognitive function. Items are scored as correct or incorrect, with one point assigned for each correct response, for a total score from 0 to 10. Scores of 0 to 2 indicate intact cognitive function, 3 to 4 indicate mild cognitive impairment, and 5 to 10 indicate moderate to severe cognitive impairment. Score interpretation is adjusted for education level. Cronbach’s alpha in this study was 0.722.

### 2.5 Data collection

Field surveys were conducted from January to June 2020 in six regions of Sichuan Province, China: Deyang, Chengdu, Luzhou, Neijiang, Dazhou, and the Aba Tibetan and Qiang Autonomous Prefecture. These sites were selected using a multi-stage, stratified convenience sampling approach based on per capita GDP levels reported in the 2018 Sichuan Statistical Yearbook. Investigators from medical universities received standardized training to ensure consistent questionnaire administration and reduce interviewer bias. With support from local village doctors and community staff, they carried out community-based household surveys. Before data collection, investigators explained the study objectives, procedures, and participants’ right to refuse participation or withdraw at any time without consequence. Surveys were administered only after written informed consent was obtained. Given limited literacy among many rural older adults, trained interviewers conducted face-to-face interviews, read each question aloud, and recorded responses on paper questionnaires, which were later entered into an electronic database. Interviews lasted approximately 20 to 30 min, depending on response pace and the need for clarification. After each interview, questionnaires were reviewed for completeness and internal consistency. Incomplete questionnaires were addressed on site by recontacting participants when needed, and questionnaires with substantial missing data, defined as more than 10% of items unanswered, were excluded from analysis. Owing to on-site review and clarification, missing data in the final dataset were minimal, at less than 5% across variables. Additional quality-control procedures included logical checks of responses and, when applicable, cross-verification with community staff. All data were anonymized and used solely for academic research.

### 2.6 Statistical methods

Analyses were performed using IBM SPSS Statistics version 24.0 (IBM Corp., Armonk, NY, United States) and Mplus version 8.3 (Muthén and Muthén, Los Angeles, CA, USA). LPA was used to identify subgroups of ESN among older adults with chronic diseases, using the 14 SESN items as indicators. The dependent variable was latent profile membership. Model fit was assessed using the Akaike Information Criterion (AIC), Bayesian Information Criterion (BIC), adjusted BIC (aBIC), entropy, the Lo–Mendell–Rubin adjusted likelihood ratio test (LMRT), and the bootstrap likelihood ratio test (BLRT). Lower AIC, BIC, and aBIC values indicated better fit. Entropy ranges from 0 to 1, with values closer to 1 indicating higher classification accuracy. A significant LMRT or BLRT result (*p* ≤ 0.05) supported a k-class model over a k−1 class model. The final number of classes also considered interpretability and adequate class sizes. Continuous variables are presented as medians with interquartile ranges (IQR), and categorical variables as frequencies and percentages. Between-class comparisons used chi-square (*χ^2^*) tests or Kruskal–Wallis H tests, as appropriate. Multinomial logistic regression was conducted to examine factors associated with profile membership. Predictors included age, sex, education level, living alone, widowed status, self-rated economic status, grandchild caregiving, frequency of communication with children, perceived loneliness, personality, physical examination status, mobile phone use, sedentary behavior, sleep quality, self-rated health, cognitive impairment, smoking, alcohol consumption, pain, comorbidity status, depression, and social support score. Variables significant in univariate analyses were entered into multivariable logistic regression models to identify predictors of latent profile membership. Statistical significance was set at a two-tailed *p* < 0.05.

### 2.7 Ethical statement

The study protocol was reviewed and approved by the Ethics Committee of the Affiliated Hospital of Southwest Medical University (Approval No. KY2019274) and conducted in accordance with the Declaration of Helsinki. All participants provided written informed consent after a detailed explanation of the study objectives. Data were handled confidentially, and all identifying information was removed to protect participant privacy.

## 3 Results

### 3.1 General characteristics of survey subjects

A total of 785 rural older adults with chronic diseases were invited to participate in this cross-sectional study. After exclusion of 66 questionnaires with incomplete or erroneous responses, 719 participants (91.6%) were included in the analysis. Participant sociodemographic characteristics were summarized in Supplementary Table 1. Most participants were female (*n* = 392, 54.5%), and most had a junior high school education or less (*n* = 624, 86.8%). The median age was 70.0 years (IQR, 66 to 76). The prevalence of chronic diseases was as follows: hypertension (*n* = 554, 77.1%), diabetes (*n* = 154, 21.4%), coronary heart disease (*n* = 119, 16.6%), COPD (*n* = 79, 11.0%), stroke (*n* = 68, 9.5%), and cancer (*n* = 36, 5.0%). Univariate analyses (Supplementary Table 2) indicated that ESN profile distribution differed by chronic disease type. COPD was most frequently observed in the moderate neglect profile (18.9%), whereas cancer was more frequently observed in the severe neglect profile (11.1%; both *p* < 0.05). ESN profiles did not differ significantly by hypertension, diabetes, coronary heart disease, or stroke (all *p* > 0.05).

### 3.2 Status of ESN

The median total ESN score was 9 (5 to 13). Median dimension scores were 2 (1 to 4) for medical health and care, 2 (0 to 3) for environmental sanitation and personal hygiene, 2 (1 to 3) for mental health, 1 (0 to 3) for safety, and 1 (0 to 2) for social communication.

### 3.3 Potential profiles of ESN and characteristics by profile

LPA was conducted to identify subgroups of ESN among rural older adults with chronic diseases. As shown in Table 1, model fit was evaluated using the AIC, BIC, aBIC, the entropy, the LMRT, and the BLRT. The four-class model showed the best fit, with the lowest AIC (20,622.627), BIC (20,956.811), and aBIC (20,725.015), and high entropy (0.962), indicating strong classification accuracy. The LMRT (*p* = 0.0057) and BLRT (*p* < 0.001) also favored the four-class model over the three-class solution. Accordingly, the four-class model was retained for subsequent analyses.

**Table 1:** Model fit indices for latent profile analysis models.

Model	AIC	BIC	aBIC	Entropy	LMRT (*p* value)	BLRT (*p* value)	Profile prevalence (%)
1	24126.018	24254.198	24165.290	-	-	-	-
2	22151.835	22348.684	22212.147	0.917	<0.001	<0.001	77.191/22.809
3	21134.633	21400.149	21215.983	1.000	0.3618	<0.001	35.049/52.434/12.517
4	20622.627	20956.811	20725.015	0.962	0.0057	<0.001	35.049/14.743/37.691/12.517
5	20508.422	20911.274	20631.850	0.952	0.7356	<0.001	4.033/31.015/37.413/15.021/12.517

AIC, Akaike information criterion; BIC, Bayesian information criterion; aBIC, adjusted Bayesian information criterion; LMRT, Lo–Mendell–Rubin adjusted likelihood ratio test; BLRT, bootstrap likelihood ratio test.

**Figure 1:**
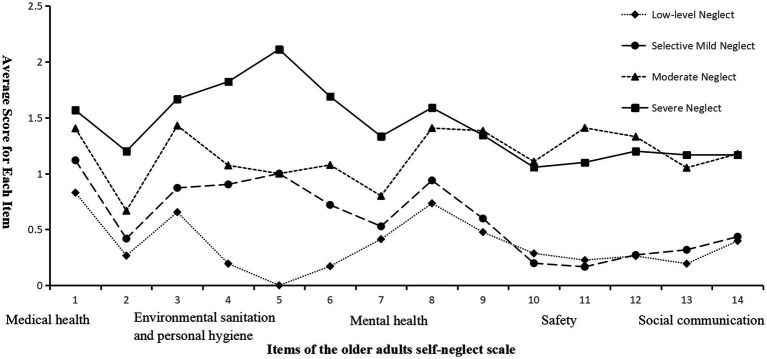
Distribution of characteristics across four latent profiles of ESN among rural older adults with chronic diseases.

**Figure 2:**
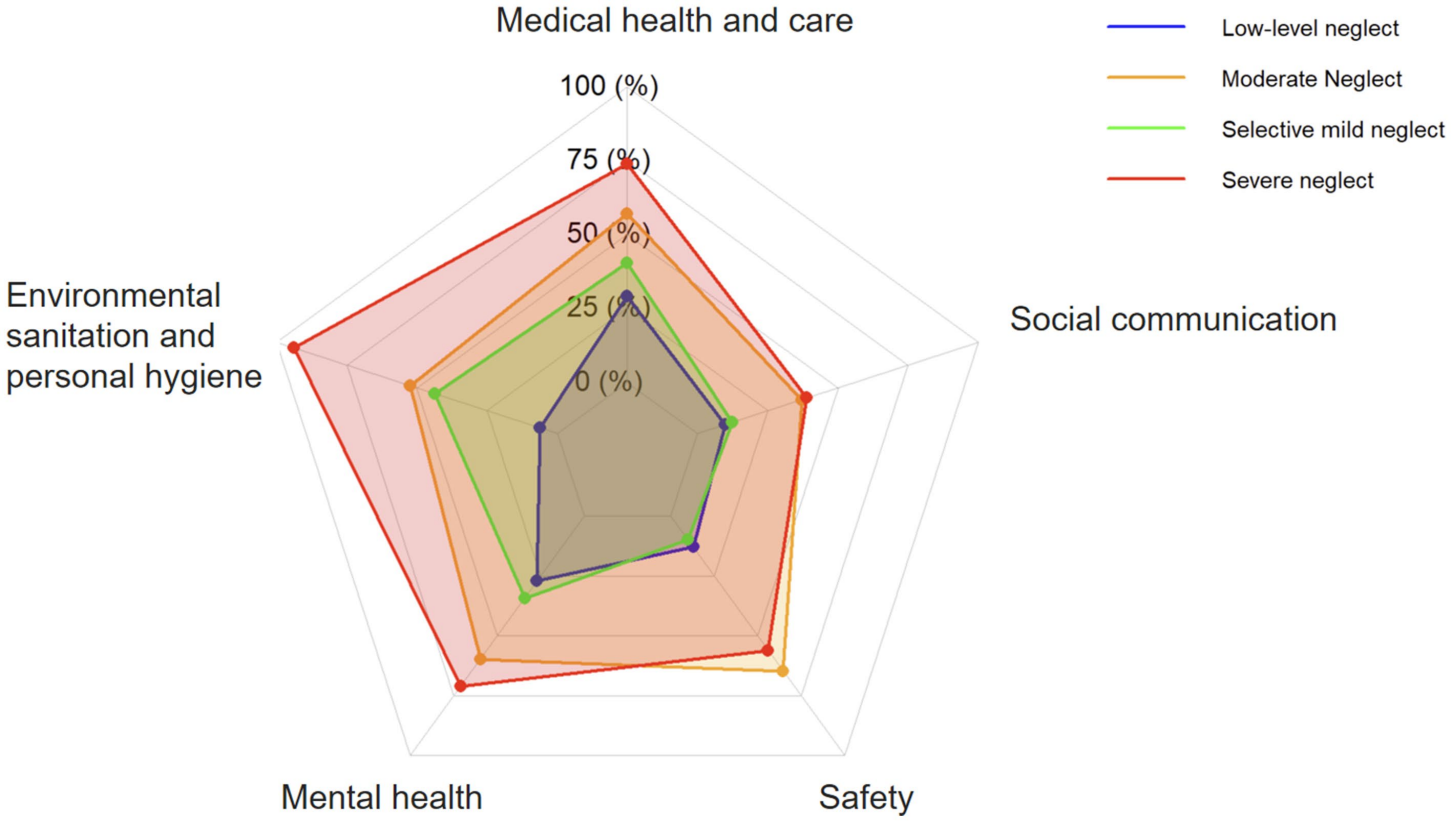
Radar chart of the five domains of ESN across four latent profiles. Each axis represents one ESN domain (medical, hygiene, psychological, safety, and social). Lines indicate mean standardized domain scores for each profile; higher scores indicate greater neglect in the corresponding domain.

Profiles were labeled to reflect an overall severity gradient and domain-specific scoring patterns on the ESN scale, aligned with the Biopsychosocial Model (36) and the medical, hygiene, psychological, safety, and social domains assessed by the instrument (Figures 1, 2). Class 1, low-level neglect (*n* = 252, 35.0%), showed consistently low scores across all 14 items. The selective mild neglect profile (*n* = 271, 37.7%) had relatively low overall scores, with comparatively higher scores in the medical and hygiene domains, indicating a selective pattern. The moderate neglect profile (*n* = 106, 14.7%) showed higher scores across domains, with particularly elevated scores in the safety, psychological, and medical domains relative to the selective mild neglect profile. The severe neglect profile (*n* = 90, 12.5%) had the highest scores across all domains. Classification quality was further examined using average latent class probabilities for most likely class membership (Supplementary Table 3). Diagonal probabilities were high (Class 1 to Class 4: 1.000, 0.916, 0.980, and 1.000), and off-diagonal probabilities were low.

Median (IQR) total ESN scores differed across profiles: 5 (2 to 7) for low-level neglect, 8 (6 to 11) for selective mild neglect, 16 (14 to 19) for moderate neglect, and 21 (14 to 25) for severe neglect (H = 422.741, *p* < 0.001). The four profiles also differed in demographic, behavioral, and psychosocial characteristics, including age, living arrangement, marital status, self-rated economic status, grandchild caregiving, frequency of communication with children, perceived loneliness, personality traits, physical examination status, mobile phone use, sedentary behavior, sleep quality, self-rated health, cognitive impairment, smoking, alcohol use, pain, depression, and social support (all *p* < 0.05; Supplementary Table 1). Participants in the moderate neglect group were older, with a median age of 74 years compared with 69 years in the low-level neglect group (*p* = 0.001). The proportion living alone increased across profiles, from 11.9% in the low-level neglect group to 30.0% in the severe neglect group (*p* < 0.001). Psychosocial vulnerability also increased, with perceived loneliness rising from 25.4% in the low-level group to 64.4% in the severe neglect group and depressive symptoms increasing from 13.5 to 54.4% (both *p* < 0.001). Poor self-rated economic status was more common in higher-risk profiles, reported by 37.8% of the severe neglect group versus 9.5% of the low-level group. Social support differed by profile (*p* < 0.001), with lower median scores in the moderate and severe neglect groups (both 37) than in the low-level neglect group.

### 3.4 Multivariate logistic regression analysis of factors influencing ESN profiles

No multicollinearity was detected among the independent variables (all tolerance values > 0.60; all VIFs < 2.0). The low-level neglect group (Class 1) was used as the reference category, and results are shown in Table 2. Compared with the low-level neglect group, participants in the moderate neglect group (Class 3) were older (OR = 1.046, *p* = 0.025) and had lower social support scores (OR = 0.924, *p* < 0.001). For family interaction, communicating with children 1–3 times a week (OR = 3.382, *p* = 0.011) or once a month (OR = 2.068, *p* = 0.016) was associated with higher odds of Class 3 membership than communicating less than once per month, whereas frequent communication was not significant. Annual physical examinations were inversely associated with moderate neglect relative to no regular examinations (OR = 0.431, *p* = 0.047). Absence of cognitive impairment, non-smoking, and absence of pain were each associated with lower odds of Class 3 membership (all *p* < 0.05), consistent with a comparatively healthier profile among those remaining in the low-level neglect group. Compared with the low-level neglect group, the severe neglect group (Class 4) showed a distinct psychosocial and behavioral pattern. Fair self-rated economic status was associated with lower odds of severe neglect relative to poor economic status (OR = 0.466, *p* = 0.047). Not providing grandchild caregiving was associated with higher odds of Class 4 membership (OR = 2.147, *p* = 0.012). Absence of perceived loneliness (OR = 0.472, *p* = 0.031), non-smoking (OR = 0.424, *p* = 0.013), and absence of depressive symptoms (OR = 0.340, *p* = 0.002) were each associated with lower odds of severe neglect.

**Table 2:** Multinomial logistic regression of factors associated with ESN profiles (*n* = 719).

Variables	Category	*β*	Standard error	Wald *χ*^2^ value	OR	95% CI	*p* value
C2 vs. C1							
Self-rated economic status	Good	−1.456	0.406	12.881	0.233	0.105–0.516	<0.001
	Fair	−0.707	0.299	5.573	0.493	0.274–0.887	0.018
	Poor	Ref.			1.000 (Ref.)	Ref.	
Sleep quality	Good	−0.690	0.329	4.395	0.502	0.263–0.956	0.036
	Fair	−0.327	0.287	1.296	0.721	0.411–1.266	0.255
	Poor	Ref.			1.000 (Ref.)	Ref.	
Alcohol	No	−0.525	0.243	4.666	0.592	0.368–0.953	0.031
	Yes	Ref.			1.000 (Ref.)	Ref.	
C3 vs. C1							
Age	-	0.045	0.020	5.007	1.046	1.006–1.088	0.025
Social Support Score	-	−0.079	0.021	13.783	0.924	0.887–0.964	<0.001
Communication with children	>3 times/week	0.029	0.579	0.002	1.029	0.331–3.204	0.960
	1–3 times/ week	1.218	0.478	6.497	3.382	1.325–8.629	0.011
	once a month	0.727	0.301	5.828	2.068	1.146–3.730	0.016
	less than once per month	Ref.			1.000 (Ref.)	Ref.	
Physical examination status	Annual	−0.842	0.425	3.929	0.431	0.187–0.991	0.047
	Occasional	−0.018	0.323	0.003	0.982	0.521–1.849	0.955
	None	Ref.			1.000 (Ref.)	Ref.	
Cognitive impairment	No	−0.716	0.359	3.978	0.489	0.242–0.988	0.046
	Yes	Ref.			1.000 (Ref.)	Ref.	
Smoking	No	−0.715	0.337	4.500	0.489	0.253–0.947	0.034
	Yes	Ref.			1.000(Ref.)	Ref.	
Pain	No	−0.743	0.322	5.328	0.475	0.253–0.894	0.021
	Yes	Ref.			1.000 (Ref.)	Ref.	
C4 vs. C1							
Self-rated economic status	Good	−0.555	0.529	1.102	0.574	0.204–1.618	0.294
	Fair	−0.763	0.384	3.952	0.466	0.220–0.989	0.047
	Poor	Ref.			1.000 (Ref.)	Ref.	
Grandchild Caregiving	No	0.764	0.305	6.268	2.147	1.180–3.904	0.012
	Yes	Ref.			1.000 (Ref.)	Ref.	
Perceived loneliness	No	−0.752	0.349	4.634	0.472	0.238–0.935	0.031
	Yes	Ref.			1.000 (Ref.)	Ref.	
Smoking	No	−0.857	0.346	6.122	0.424	0.215–0.837	0.013
	Yes	Ref.			1.000 (Ref.)	Ref.	
Depression	No	−1.078	0.355	9.217	0.340	0.170–0.682	0.002
	Yes	Ref.			1.000 (Ref.)	Ref.	

Additional pairwise comparisons of adjacent profiles (Class 3 vs. Class 2, Class 4 vs. Class 2, and Class 4 vs. Class 3) identified stage-dependent associations for several factors (Supplementary Table 4). Social support reduced the odds of moderate neglect relative to selective mild neglect (OR = 0.929, *p* < 0.001) but was associated with higher odds of severe neglect relative to moderate neglect (OR = 1.060, *p* = 0.021), indicating a reversal at higher severity. Absence of pain was associated with lower odds of moderate neglect relative to selective mild neglect (OR = 0.474, *p* = 0.018) yet higher odds of severe neglect relative to moderate neglect (OR = 2.811, *p* = 0.009), consistent with a non-linear pattern.

### 3.5 Interaction analysis of internal vulnerabilities and external risks

Guided by the Risk and Vulnerability Model of self-neglect (37), interaction terms were added to the multivariable multinomial logistic regression models to assess whether internal vulnerabilities and external risk factors jointly influenced ESN latent profile membership. Five interactions were examined: living alone × cognitive impairment, social support × depression, age × pain, age × social support score, and age × self-rated health. Among these, only the age × pain interaction reached statistical significance. Relative to the low-level neglect group (Class 1), the age × pain interaction was significant for selective mild neglect (OR = 1.063, *p* = 0.043) and was stronger for moderate neglect (OR = 1.124, *p* = 0.016). These findings indicate that the relationship between physical pain and ESN profile membership varies by age. No other interaction terms were significant across group comparisons (all *p* > 0.05). Detailed results are provided in Supplementary Table 5.

## 4 Discussion

### 4.1 Interpretation of the four profiles

LPA identified four subtypes of ESN among rural Chinese older adults with chronic diseases, supporting the heterogeneity of ESN. The four profiles, low-level (35.0%), selective mild (37.7%), moderate (14.7%), and severe (12.5%), differed in overall severity and in domain-specific patterns across the five SESN domains (medical health and care, environmental sanitation and personal hygiene, mental health, safety, and social communication; Figures 1, 2). The low-level neglect profile showed consistently low scores across domains, suggesting strong engagement in self-care and relatively intact biopsychosocial resources. Individuals in this group may have sufficient agency to meet daily and health-related demands. In the context of Orem’s Self-Care Deficit Nursing Theory (38), this profile may represent a domain-specific self-care deficit in which therapeutic self-care demands related to chronic illness, such as medication management, diet, and hygiene routines, exceed self-care agency in selected areas, while other domains remain preserved or compensated. The moderate neglect profile showed broader elevations, particularly in the safety, psychological, and medical domains, consistent with progressive accumulation of deficits in the presence of older age, pain, cognitive impairment, and lower social support. The severe neglect profile showed the highest scores across all domains, indicating pervasive disruption of self-care systems and the greatest health vulnerability.

This typology aligns in part with the work of Burnett et al. (25), who applied latent class analysis to U.S. APS data and identified four subtypes: predominant physical and medical neglect (48%), environmental neglect (22%), global or multidimensional neglect (21%), and financial neglect (9%). Both studies indicate substantial heterogeneity in ESN and a large subgroup with prominent physical and medical impairments. The present findings also add context-specific detail. The selective mild profile emphasizes discrete difficulties in chronic disease self-management, including medical compliance and hygiene, rather than financial neglect or severe environmental problems described in APS-referred samples. These differences may reflect the community-based rural Chinese sample with diagnosed chronic conditions, where disease-related demands and limited access to care may heighten specific vulnerabilities. Together, the profiles suggest that ESN follows distinct patterns, offering a context-relevant classification to inform targeted interventions.

### 4.2 The influencing factors of latent classes of ESN in older adults with chronic diseases

#### 4.2.1 Sociodemographic factors

Age was significantly related to ESN profile membership. In the comparison of moderate neglect versus low-level neglect, each additional year of age corresponded to a 4.6% increase in the odds of moderate neglect (OR = 1.046, *p* = 0.025). This pattern is consistent with the vulnerability accumulation hypothesis in geriatric research (39–41). With advancing age, declines in physical function, mobility, and sensory capacity, together with multimorbidity, may coincide with reduced self-care capacity. In rural settings, aging may also coincide with smaller social networks and role loss, which may be linked to lower motivation to maintain hygiene and living conditions and, in turn, to higher levels of self-neglect (42). Chronic pain may further coincide with limitations in mobility and daily functioning, and this relationship may be more evident in rural areas with limited external support and health care resources (40). The significant age-by-pain interaction observed in this study indicates that the association between pain and ESN profile membership varies by age. Prior work suggests that vulnerability to chronic pain increases with age, and that the combined burden of pain and disability is associated with worse health outcomes (43). These findings support prioritizing older adults aged 75 years and above in primary care, with attention to functional support and reinforcement of social support structures.

Self-rated economic status showed stage-dependent associations with self-neglect, using the low-level neglect group (Class 1) as the reference. In the selective mild neglect group (Class 2), good economic status was associated with 76.7% lower odds of selective mild neglect (OR = 0.233, *p* < 0.001), consistent with evidence that better economic conditions are linked to greater access to care and social participation (44, 45). In the severe neglect group (Class 4), fair economic status remained associated with lower odds relative to poor status (OR = 0.466, *p* = 0.047), whereas good economic status was not statistically significant (*p* = 0.294). These results suggest that severe economic hardship may be more closely linked to severe self-neglect, while higher economic standing alone may not distinguish the most advanced profiles. In rural China, this pattern suggests that financial subsidies may be more relevant for addressing milder neglect, whereas severe ESN may call for approaches that combine material assistance with mental health support.

#### 4.2.2 Physical health status and health behaviors

Health-compromising behaviors, including smoking and alcohol consumption, were associated with greater ESN severity. Compared with the low-level neglect group (Class 1), non-smoking was associated with lower odds of moderate neglect (Class 3 vs. Class 1: OR = 0.489, *p* = 0.034) and severe neglect (Class 4 vs. Class 1: OR = 0.424, *p* = 0.013). Alcohol abstinence was also associated with lower odds of selective mild neglect (Class 2 vs. Class 1: OR = 0.592, *p* = 0.031). Avoidance of smoking and drinking may reflect stronger self-efficacy and greater engagement in health maintenance, which may coincide with lower levels of self-neglect. In rural China, smoking and drinking are often used to cope with psychological distress, loneliness, or chronic pain. These behaviors may be linked to lower engagement in proactive self-care and long-term health management (46). Prior studies also suggest that long-term tobacco and alcohol use, particularly through effects on cognitive function, may relate to difficulties in daily functioning and adherence to medical regimens (47).

Pain showed stage-specific associations with ESN profiles. Older adults without pain had lower odds of moderate neglect than low-level neglect and selective mild neglect (Class 3 vs. Class 1: OR = 0.475, *p* = 0.021; Class 3 vs. Class 2: OR = 0.474, *p* = 0.018). In rural settings, pain may be linked to reduced mobility and energy and may be associated with challenges in self-care. Pain has also been related to cognitive functioning and health-related decision-making (48), which may help explain broader difficulties beyond medical adherence. In contrast, the absence of pain was associated with higher odds of severe neglect than moderate neglect (Class 4 vs. Class 3: OR = 2.811, *p* = 0.009). One possible explanation is that pain is a salient symptom that may draw attention from family or neighbors, whereas absence of pain in the severe neglect profile may reflect reduced symptom awareness or under-reporting, potentially related to apathy or cognitive impairment (49). Absence of pain also does not necessarily indicate low disease burden. Among older adults with chronic conditions, reduced pain perception may reflect sensory impairment or neuropathy, such as diabetic peripheral neuropathy, which can blunt warning signals and delay help-seeking, and may be associated with severe, unrecognized neglect. Consistent with this interpretation, the higher concentration of COPD and cancer in more advanced neglect profiles aligns with the potential role of symptom burden and systemic frailty in self-care difficulties.

Sleep quality was also associated with ESN. Good sleep quality was associated with lower odds of self-neglect than poor sleep quality (OR = 0.502, *p* = 0.036), consistent with Yunus et al. (50). Sleep is closely related to cognitive and physical function, including executive function (51), which may affect tasks such as medication management and maintaining environmental hygiene. Chronic sleep deprivation has been associated with fatigue and cognitive decline, which may coincide with reduced engagement in self-care (52). The absence of a significant association for fair sleep quality suggests that the pattern may be most evident at the extremes. Sleep hygiene may therefore warrant consideration in health promotion for this population.

Physical examination status was associated with ESN profiles. Annual physical examinations were associated with lower odds of self-neglect compared with never undergoing screenings (OR = 0.431, *p* = 0.047), whereas occasional examinations were not significant (*p* = 0.955). Annual examinations may indicate greater health consciousness and more consistent engagement with health services (53). In rural areas, regular examinations may also offer opportunities for monitoring and for recognizing problems related to self-care, supporting timely follow-up by health care providers and family members. The lack of a significant association for occasional screening suggests that more consistent engagement with formal health services may be more closely linked to sustained self-care.

#### 4.2.3 Social factors

Several social variables showed stage-dependent or non-linear associations with ESN profiles, pointing to complex links across psychosocial and behavioral domains. Social support showed a reversal. Higher support scores were associated with lower odds of moderate neglect compared with low-level neglect (Class 3 vs. Class 1: OR = 0.924, *p* < 0.001) and selective mild neglect (Class 3 vs. Class 2: OR = 0.929, *p* < 0.001). In contrast, higher support scores were associated with higher odds of severe neglect compared with moderate neglect (Class 4 vs. Class 3: OR = 1.060, *p* = 0.021). This pattern may reflect stage-related differences in the form and function of support rather than adverse effects of support (54). With greater ESN severity, instrumental assistance may be more frequently reported and may coincide with reduced personal agency and greater dependency (10, 12). Interpretation is limited by the SSRS, which assesses objective, subjective, and utilization components but does not distinguish instrumental from emotional support. The observed reversal is consistent with the Chronic Stress Model (55), in which compensatory networks may become more prominent after substantial deterioration and may co-occur with lower self-efficacy.

In rural China, family caregiving is widespread, but emotional and informal support may be insufficient to sustain self-care autonomy in later ESN stages (54). Resource constraints may also be associated with greater reliance on external support. In advanced stages, community-based services may have limited coordination and capacity for complex needs. These observations support attention to support quality in earlier stages and, in later stages, to approaches that balance supervision with autonomy support.

Grandchild caregiving also differed by profile. Older adults not engaged in grandchild caregiving had higher odds of severe neglect than those in the low-level group (OR = 2.147) and the moderate group (OR = 2.027). This pattern is consistent with the active aging framework, which links engagement in productive family roles with better self-care (56). In rural settings, caregiving may reflect social engagement and reciprocity and may coincide with greater practical support and health-related monitoring from adult children. At the same time, high caregiving demands may be associated with role strain, fatigue, and time scarcity that could relate to reduced self-care. Because caregiving intensity and burden were not measured, future studies should assess whether this association is non-linear and varies by caregiving demands.

Communication frequency with children showed another non-linear pattern. Moderate contact (“1–3 times/week” or “once a month”) was associated with higher odds of moderate neglect relative to low-level neglect (ORs 2.068 to 3.382), which may reflect contact that does not address underlying needs (57). Occasional communication was associated with lower odds of severe neglect relative to moderate neglect (Class 4 vs. Class 3: OR = 0.406, *p* = 0.023), suggesting that limited contact may still coincide with monitoring and a safety-net function in rural contexts. This interpretation is consistent with Socioemotional Selectivity Theory (58), which emphasizes emotionally meaningful contact; infrequent but responsive communication may be associated with timely support.

#### 4.2.4 Psychological and cognitive factors

Depression and loneliness showed the strongest and most consistent associations with severe self-neglect (Class 4). Absence of depressive symptoms was associated with lower odds of severe neglect across comparisons (Class 4 vs. Class 1: OR = 0.340, *p* = 0.002; Class 4 vs. Class 2: OR = 0.474, *p* = 0.025; Class 4 vs. Class 3: OR = 0.470, *p* = 0.049), corresponding to approximately 52.6 to 66.0% lower odds. Absence of perceived loneliness was likewise associated with lower odds of severe neglect in key pairwise models (Class 4 vs. Class 1: OR = 0.472, *p* = 0.031; Class 4 vs. Class 2: OR = 0.385, *p* = 0.005). These associations may reflect linked psychological and biological processes. Depression often involves apathy, anhedonia, and impaired emotional regulation, which may coincide with reduced engagement in essential self-care. Neuropsychological research has also linked depression to reduced prefrontal inhibitory control and executive dysfunction, which may relate to difficulties in decision-making and behavioral initiation (59). Loneliness represents subjective social disconnection that may erode self-worth and self-efficacy. Both depression and loneliness have been linked to hypothalamic–pituitary–adrenal axis activation, sustained cortisol elevation, neuroinflammation, and neural degradation, which may coincide with emotional dysregulation and reduced motivation for self-care (60). Among rural older adults with chronic diseases, these processes may co-occur with high treatment demands and limited support and may be more common in severe neglect profiles. Incorporating brief screening for depressive symptoms and loneliness into routine chronic disease management in rural primary care may help identify older adults with a higher likelihood of severe ESN. Positive screens could prompt resource-adapted psychosocial support and referral for further assessment and treatment when indicated. Longitudinal studies are needed to clarify temporal relationships and to assess whether screening and follow-up are associated with less worsening of self-neglect over time.

Cognitive impairment also differentiated profiles. Absence of cognitive impairment was associated with lower odds of moderate neglect compared with low-level neglect (OR = 0.489, *p* = 0.046) and selective mild neglect (OR = 0.444, *p* = 0.015), corresponding to an approximate 51 to 56% reduction in odds. Even mild deficits may affect executive functions needed for treatment adherence, hazard recognition, and hygiene maintenance (61). In rural settings with limited oversight, impaired judgment and memory may be less readily detected and may coincide with higher levels of neglect (62). Brief cognitive screening alongside depression and loneliness assessments during community health visits may support earlier recognition. Strategies such as cognitive stimulation activities, family supervision, medication reminders, or digital aids may help maintain remaining self-care capacity and may be relevant for individuals with more severe ESN profiles.

Taken together, correlates of ESN profiles indicate an interrelated risk structure in which physical vulnerability (pain and cognitive impairment), psychosocial distress (depression and loneliness), and social resources (social support and family role engagement) may cluster and influence self-care capacity across stages. The significant age by pain interaction further suggests that the implications of pain for ESN risk vary by age. In addition, several social indicators exhibited non-monotonic patterns across adjacent profiles, indicating that risk factors may not operate uniformly across severity levels. Accordingly, these results support a stage-specific interpretation rather than a single linear risk gradient.

### 4.3 Practical implications

The heterogeneous ESN profiles identified in this study suggest a tiered, community-based approach in rural China. Class 4 represents the highest-risk group, with pervasive deficits and strong associations with depressive symptoms, loneliness, smoking, economic hardship, and lack of grandchild caregiving roles. Class 3 represents the second-highest risk group and shows broader impairments, particularly in the safety, psychological, and medical domains; it was associated with older age, pain, cognitive impairment, lower social support, and infrequent physical examinations. Prioritizing these high-risk subgroups may support more efficient targeting in resource-constrained rural settings.

Selective mild neglect may serve as an early focus, with attention to modifiable lifestyle and resource vulnerabilities. Village-level coordination could include strengthened financial protection for chronic disease management and targeted health education addressing sleep problems and alcohol use. For moderate neglect, township health centers could implement routine monitoring through home visits or telephone follow-ups that include basic health checks, pain assessment, brief cognitive screening, and chronic disease self-management support. For severe neglect, a multidisciplinary approach may be considered, with emphasis on scalable psychosocial support and loneliness reduction, along with caregiver support and respite services where available. Screening for depressive symptoms, loneliness, and cognitive impairment could be incorporated into routine chronic disease management to identify older adults more likely to be classified in the severe ESN profile, while noting that longitudinal studies are needed to confirm transitions between profiles.

At the policy level, these findings may inform national rural health initiatives, including the National Basic Public Health Service Program (NBPHSP) and the Healthy China 2030 strategy, by incorporating ESN risk stratification and profile-informed service packages. Directing services toward depressed and lonely individuals, those with cognitive impairment or pain, smokers, economically disadvantaged older adults, and those with limited engagement in family roles may support more targeted allocation within constrained rural health infrastructure.

This study has several limitations. First, the cross-sectional design captures associations at a single time point and does not allow causal inference. Second, self-reported questionnaires may be subject to recall or social desirability bias. In addition, although the Chinese versions of the UCLALS-3, GDS-5, and SPMSQ have been widely used in older Chinese adults with acceptable reliability, including in the current sample, psychometric validation specifically in rural Chinese older populations is limited. Lower literacy and differences in cultural expression of emotional distress may affect measurement accuracy for loneliness, depression, and cognitive impairment. Third, the study did not include several environmental and systemic factors, including family caregiving intensity, access to community health resources, and medical insurance coverage. In rural China, these factors influence individuals’ ability to manage health and seek care, and their omission may limit the completeness of the identified profiles and their predictors. Data collection occurred from January to June 2020, during the early stage of the COVID-19 pandemic; although study areas were not high-risk regions, the pandemic may have influenced health care seeking, social support, and psychological status. Finally, convenience sampling within one rural province and the focus on six chronic diseases may limit generalizability to other regions or broader disease groups.

Future studies should use longitudinal designs to examine transitions between ESN profiles over time, clarify temporal relationships, and identify time points for earlier intervention. Qualitative interviews with individuals in each profile and their caregivers could provide insight into lived experience, barriers to self-care, and contextual factors related to heterogeneity. Randomized controlled trials tailored to each profile, including economic and lifestyle support for selective mild neglect, regular monitoring and health literacy programs for moderate neglect, and intensive psychosocial interventions for severe neglect, are needed to evaluate feasibility and efficacy in rural settings. Incorporating biological markers, such as inflammatory indicators and cortisol levels, or functional measures may further clarify mechanisms related to profile stability.

## 5 Conclusion

This study demonstrates substantial heterogeneity in ESN among rural older adults living with chronic diseases. Four profiles were identified: low-level neglect, selective mild neglect, moderate neglect, and severe neglect. Risk factors varied across profiles, with external stressors more salient in the lower-severity profiles and internal vulnerabilities increasingly prominent as neglect severity increased. Selective mild neglect clustered with economic and lifestyle challenges, including financial insecurity, inadequate sleep, and alcohol consumption. In the moderate neglect profile, functional decline and social vulnerability were more evident; older age, pain, cognitive decline, and low social support emerged as key contributors. Severe neglect showed the strongest links to psychological distress, particularly depression and loneliness, alongside diminished engagement in family roles, most notably the absence of grandchild caregiving. These findings support a profile-informed, tiered approach to managing ESN in rural primary care and community health services. For the selective mild neglect profile, priorities include strengthening financial protection for chronic disease management and providing targeted education on sleep hygiene and alcohol use. For the moderate neglect profile, township health centers could deliver routine follow-up (home visits or telephone contacts) incorporating basic health checks, pain assessment, brief cognitive screening, and self-management support. For the severe neglect profile, services should prioritize timely screening and referral for depression and loneliness, together with scalable psychosocial support and, where feasible, support for maintaining caregiver or family role engagement. Integrating ESN risk stratification and profile-specific service packages into established rural programs (e.g., NBPHSP and Healthy China 2030) may improve targeting under resource constraints.

## Data Availability

The raw data supporting the conclusions of this article will be made available by the authors, without undue reservation.
