# Supplementary material for: Latent profile analysis of self-neglect and associated factors among rural older adults with chronic diseases: a cross-sectional study
Source: Front Public Health. 2026 Jan 28;14:1738418. doi: 10.3389/fpubh.2026.1738418 (PMC12897509; doi:10.3389/fpubh.2026.1738418)
Supplement: Supplementary file 1 [file Table_1.docx]

Supplementary Material

**Supplementary Table 1. Demographic information of survey subjects and univariate analysis of potential profiles of ESN (n = 719).**

| **Characteristic** | **Overall  N = 719** | **Low-level neglect n1 = 252** | **Selective mild neglect n2 = 271** | **Moderate neglect  n3 = 106** | **Severe Neglect  n4 = 90** | ***Kruskal-Wallis H/χ^2^*** | ***p* value** |
| --- | --- | --- | --- | --- | --- | --- | --- |
| Age | 70.00 (66.00, 76.00) | 69.00 (66.00, 74.00) | 70.00 (66.00, 76.00) | 74.00 (68.00, 78.00) | 70.00 (66.00, 77.00) | 15.532^a^ | 0.001 |
| Sex |  |  |  |  |  | 7.078^b^ | 0.069 |
| Female | 392 (54.5%) | 146 (57.9%) | 153 (56.5%) | 46 (43.4%) | 47 (52.2%) |  |  |
| Male | 327 (45.5%) | 106 (42.1%) | 118 (43.5%) | 60 (56.6%) | 43 (47.8%) |  |  |
| Education |  |  |  |  |  | 6.670^b^ | 0.083 |
| Less than junior high school | 624(86.8%) | 208(82.5%) | 243(89.7%) | 95(89.6%) | 78(86.7%) |  |  |
| Junior high  school and above | 95(13.2%) | 44(17.5%) | 28(10.3%) | 11(10.4%) | 12(13.3%) |  |  |
| Living alone |  |  |  |  |  | 17.030^b^ | <0.001 |
| No | 598 (83.2%) | 222 (88.1%) | 229 (84.5%) | 84 (79.2%) | 63 (70.0%) |  |  |
| Yes | 121 (16.8%) | 30 (11.9%) | 42 (15.5%) | 22 (20.8%) | 27 (30.0%) |  |  |
| Widowed status |  |  |  |  |  | 8.163^b^ | 0.043 |
| No | 502 (69.8%) | 191 (75.8%) | 184 (67.9%) | 72 (67.9%) | 55 (61.1%) |  |  |
| Yes | 217 (30.2%) | 61 (24.2%) | 87 (32.1%) | 34 (32.1%) | 35 (38.9%) |  |  |
| Self-rated economic status |  |  |  |  |  | 60.969^b^ | <0.001 |
| Good | 86 (12.0%) | 52 (20.6%) | 19 (7.0%) | 5 (4.7%) | 10 (11.1%) |  |  |
| Fair | 497 (69.1%) | 176 (69.8%) | 194 (71.6%) | 81 (76.4%) | 46 (51.1%) |  |  |
| Poor | 136 (18.9%) | 24 (9.5%) | 58 (21.4%) | 20 (18.9%) | 34 (37.8%) |  |  |
| Grandchild Caregiving |  |  |  |  |  | 20.334^b^ | <0.001 |
| No | 353 (49.1%) | 102 (40.5%) | 136 (50.2%) | 54 (50.9%) | 61 (67.8%) |  |  |
| Yes | 366 (50.9%) | 150 (59.5%) | 135 (49.8%) | 52 (49.1%) | 29 (32.2%) |  |  |
| Frequency of communication with children |  |  |  |  |  | 66.121^b^ | <0.001 |
| less than once per month | 385 (53.5%) | 166 (65.9%) | 147 (54.2%) | 37 (34.9%) | 35 (38.9%) |  |  |
| once a month | 209 (29.1%) | 62 (24.6%) | 81 (29.9%) | 45 (42.5%) | 21 (23.3%) |  |  |
| 1–3 times/ week | 71 (9.9%) | 11 (4.4%) | 27 (10.0%) | 17 (16.0%) | 16 (17.8%) |  |  |
| >3 times/week | 54 (7.5%) | 13 (5.2%) | 16 (5.9%) | 7 (6.6%) | 18 (20.0%) |  |  |
| Perceived loneliness |  |  |  |  |  | 51.900^b^ | <0.001 |
| No | 458 (63.7%) | 188 (74.6%) | 183 (67.5%) | 55 (51.9%) | 32 (35.6%) |  |  |
| Yes | 261 (36.3%) | 64 (25.4%) | 88 (32.5%) | 51 (48.1%) | 58 (64.4%) |  |  |
| Personality |  |  |  |  |  | 13.532^b^ | 0.035 |
| Introverted | 144 (20.0%) | 43 (17.1%) | 52 (19.2%) | 20 (18.9%) | 29 (32.2%) |  |  |
| Neutral | 309 (43.0%) | 110 (43.7%) | 112 (41.3%) | 54 (50.9%) | 33 (36.7%) |  |  |
| Extroverted | 266 (37.0%) | 99 (39.3%) | 107 (39.5%) | 32 (30.2%) | 28 (31.1%) |  |  |
| Physical examination status |  |  |  |  |  | 21.371^b^ | 0.002 |
| Annual physical examination | 177 (24.6%) | 79 (31.3%) | 65 (24.0%) | 13 (12.3%) | 20 (22.2%) |  |  |
| Occasional physical examination | 371 (51.6%) | 131 (52.0%) | 132 (48.7%) | 61 (57.5%) | 47 (52.2%) |  |  |
| No physical examination | 171 (23.8%) | 42 (16.7%) | 74 (27.3%) | 32 (30.2%) | 23 (25.6%) |  |  |
| Mobile phone use |  |  |  |  |  | 34.648^b^ | <0.001 |
| Smartphone | 131 (18.2%) | 69 (27.4%) | 39 (14.4%) | 13 (12.3%) | 10 (11.1%) |  |  |
| Basic mobile Phone | 451 (62.7%) | 148 (58.7%) | 185 (68.3%) | 67 (63.2%) | 51 (56.7%) |  |  |
| Does not use mobile phone | 137 (19.1%) | 35 (13.9%) | 47 (17.3%) | 26 (24.5%) | 29 (32.2%) |  |  |
| Sedentary behavior |  |  |  |  |  | 15.731^b^ | 0.001 |
| No | 393 (54.7%) | 150 (59.5%) | 159 (58.7%) | 48 (45.3%) | 36 (40.0%) |  |  |
| Yes | 326 (45.3%) | 102 (40.5%) | 112 (41.3%) | 58 (54.7%) | 54 (60.0%) |  |  |
| Sleep quality |  |  |  |  |  | 18.610^b^ | 0.005 |
| Good | 205 (28.5%) | 91 (36.1%) | 64 (23.6%) | 29 (27.4%) | 21 (23.3%) |  |  |
| Fair | 386 (53.7%) | 129 (51.2%) | 155 (57.2%) | 58 (54.7%) | 44 (48.9%) |  |  |
| Poor | 128 (17.8%) | 32 (12.7%) | 52 (19.2%) | 19 (17.9%) | 25 (27.8%) |  |  |
| Self-rated health |  |  |  |  |  | 31.126^b^ | <0.001 |
| Good | 111 (15.4%) | 57 (22.6%) | 26 (9.6%) | 15 (14.2%) | 13 (14.4%) |  |  |
| Fair | 443 (61.6%) | 149 (59.1%) | 186 (68.6%) | 65 (61.3%) | 43 (47.8%) |  |  |
| Poor | 165 (22.9%) | 46 (18.3%) | 59 (21.8%) | 26 (24.5%) | 34 (37.8%) |  |  |
| Cognitive impairment |  |  |  |  |  | 21.580^b^ | <0.001 |
| No | 616 (85.7%) | 227 (90.1%) | 239 (88.2%) | 77 (72.6%) | 73 (81.1%) |  |  |
| Yes | 103 (14.3%) | 25 (9.9%) | 32 (11.8%) | 29 (27.4%) | 17 (18.9%) |  |  |
| Smoking |  |  |  |  |  | 24.961^b^ | <0.001 |
| No | 528 (73.4%) | 202 (80.2%) | 208 (76.8%) | 64 (60.4%) | 54 (60.0%) |  |  |
| Yes | 191 (26.6%) | 50 (19.8%) | 63 (23.2%) | 42 (39.6%) | 36 (40.0%) |  |  |
| Alcohol |  |  |  |  |  | 8.026^b^ | 0.045 |
| No | 504 (70.1%) | 192 (76.2%) | 186 (68.6%) | 67 (63.2%) | 59 (65.6%) |  |  |
| Yes | 215 (29.9%) | 60 (23.8%) | 85 (31.4%) | 39 (36.8%) | 31 (34.4%) |  |  |
| Pain |  |  |  |  |  | 17.591^b^ | <0.001 |
| No | 240 (33.4%) | 104 (41.3%) | 89 (32.8%) | 20 (18.9%) | 27 (30.0%) |  |  |
| Yes | 479 (66.6%) | 148 (58.7%) | 182 (67.2%) | 86 (81.1%) | 63 (70.0%) |  |  |
| Comorbidity status |  |  |  |  |  | 7.490^b^ | 0.058 |
| No | 503 (70.0%) | 179 (71.0%) | 201 (74.2%) | 65 (61.3%) | 58 (64.4%) |  |  |
| Yes | 216 (30.0%) | 73 (29.0%) | 70 (25.8%) | 41 (38.7%) | 32 (35.6%) |  |  |
| Depression |  |  |  |  |  | 65.543^b^ | <0.001 |
| No | 538 (74.8%) | 218 (86.5%) | 210 (77.5%) | 69 (65.1%) | 41 (45.6%) |  |  |
| Yes | 181 (25.2%) | 34 (13.5%) | 61 (22.5%) | 37 (34.9%) | 49 (54.4%) |  |  |
| Social Support Score | 40 (34, 45) | 42 (36, 47) | 40 (35, 45) | 37 (30, 41) | 37 (31, 42) | 54.445^a^ | <0.001 |

^a^ Kruskal-Wallis H test; ^b^ chi-square test.

**
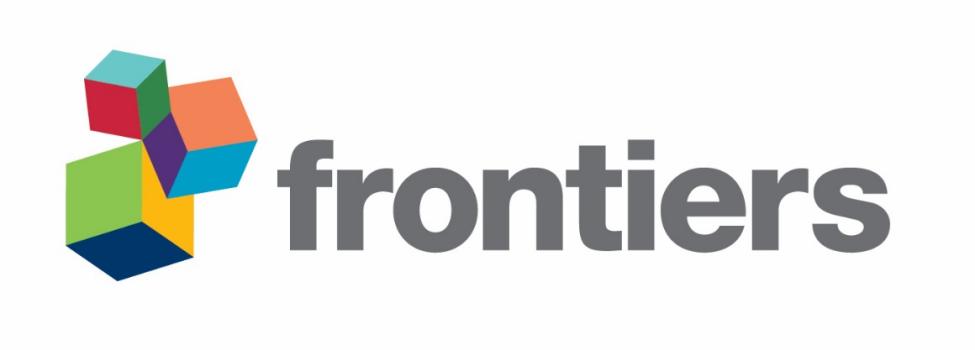
**
