# Supplementary material for: Latent profile analysis of self-neglect and associated factors among rural older adults with chronic diseases: a cross-sectional study
Source: Front Public Health. 2026 Jan 28;14:1738418. doi: 10.3389/fpubh.2026.1738418 (PMC12897509; doi:10.3389/fpubh.2026.1738418)
Supplement: Supplementary file 2 [file Table_2.docx]

Supplementary Material

**Supplementary Table 2. Distribution of ESN profiles across specific chronic disease types (n = 719).**

| **Chronic disease types** | **Overall  N = 719** | **Low-level neglect n1 = 252** | **Selective mild neglect n3 = 271** | **Moderate neglect  n2 = 106** | **Severe neglect  n4 = 90** | ***χ^2^*** | ***p* value** |
| --- | --- | --- | --- | --- | --- | --- | --- |
| Hypertension |  |  |  |  |  | 4.661 | 0.198 |
| No | 165(22.9%) | 55(21.8%) | 70(25.8%) | 17(16.0%) | 23(25.6%) |  |  |
| Yes | 554(77.1%) | 197(78.2%) | 201(74.2%) | 89(84.0%) | 67(74.4%) |  |  |
| Diabetes |  |  |  |  |  | 1.775 | 0.620 |
| No | 565(78.6%) | 197(78.2%) | 212(78.2%) | 88(83.0%) | 68(75.6%) |  |  |
| Yes | 154(21.4%) | 55(21.8%) | 59(21.8%) | 18(17.0%) | 22(24.4%) |  |  |
| Coronary heart disease |  |  |  |  |  | 2.098 | 0.552 |
| No | 600(83.4%) | 221(83.7%) | 231(85.2%) | 84(79.2%) | 74(82.2%) |  |  |
| Yes | 119(16.6%) | 41(16.3%) | 40(14.8%) | 22(20.8%) | 16(17.8%) |  |  |
| Stroke |  |  |  |  |  | 3.167 | 0.367 |
| No | 651(90.5%) | 223(88.5%) | 247(91.1%) | 100(94.3%) | 81(90.0%) |  |  |
| Yes | 68(9.5%) | 29(11.5%) | 24(8.9%) | 6(5.7%) | 9(10.0%) |  |  |
| COPD |  |  |  |  |  | 9.957 | 0.019 |
| No | 640(89.0%) | 233(92.5%) | 242(89.3%) | 86(81.1%) | 79(87.8%) |  |  |
| Yes | 79(11.0%) | 19(7.5%) | 29(10.7%) | 20(18.9%) | 11(12.2%) |  |  |
| Cancer |  |  |  |  |  | 11.466 | 0.009 |
| No | 683(95.0%) | 242(96.0%) | 263(97.0%) | 98(92.5%) | 80(88.9%) |  |  |
| Yes | 36(5.0%) | 10(4.0%) | 8(3.0%) | 8(7.5%) | 10(11.1%) |  |  |

COPD, chronic obstructive pulmonary disease; ESN, self-neglect among older adults.

**
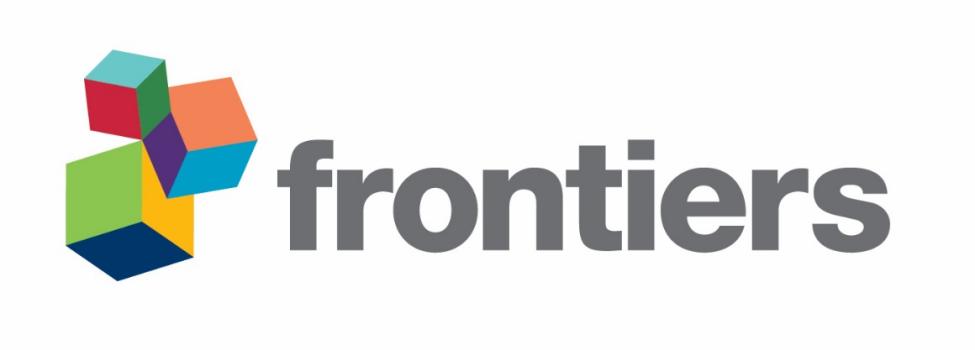
**
