# Supplementary material for: Latent profile analysis of self-neglect and associated factors among rural older adults with chronic diseases: a cross-sectional study
Source: Front Public Health. 2026 Jan 28;14:1738418. doi: 10.3389/fpubh.2026.1738418 (PMC12897509; doi:10.3389/fpubh.2026.1738418)
Supplement: Supplementary file 3 [file Table_3.docx]

Supplementary Material

**Supplementary Table 3. Average latent profiles probabilities for most likely latent class membership (row) by latent profiles (column).**

| **Most likely class** | **Class 1** | **Class 2** | **Class 3** | **Class 4** |
| --- | --- | --- | --- | --- |
| 1 | 1.000 | 0.000 | 0.000 | 0.000 |
| 2 | 0.000 | 0.916 | 0.084 | 0.000 |
| 3 | 0.000 | 0.020 | 0.980 | 0.000 |
| 4 | 0.000 | 0.000 | 0.000 | 1.000 |

Diagonal elements represent the average posterior probability for the assigned class (AvePP); off-diagonal elements reflect classification ambiguity.

**
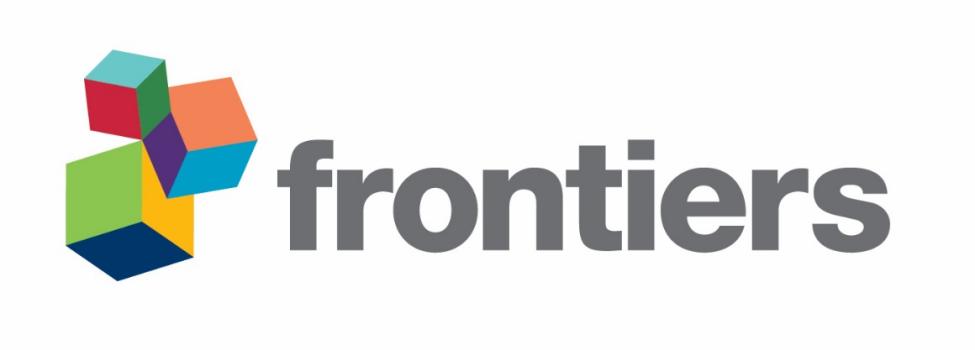
**
