# Supplementary material for: Latent profile analysis of self-neglect and associated factors among rural older adults with chronic diseases: a cross-sectional study
Source: Front Public Health. 2026 Jan 28;14:1738418. doi: 10.3389/fpubh.2026.1738418 (PMC12897509; doi:10.3389/fpubh.2026.1738418)
Supplement: Supplementary file 4 [file Table_4.docx]

Supplementary Material

**Supplementary Table 4. Pairwise multinomial logistic regression analyses between latent profiles (n = 719).**

| **Variables** | **Category** | ***β*** | **Standard error** | **Wald *χ*^2^ value** | **OR** | **95% CI** | ***p* value** |
| --- | --- | --- | --- | --- | --- | --- | --- |
| **C3 vs. C2** |  |  |  |  |  |  |  |
| Social Support Score | - | -0.074 | 0.021 | 12.825 | 0.929 | 0.892-0.967 | <0.001 |
| Widowed status | No | 0.693 | 0.343 | 4.090 | 1.999 | 1.022-3.913 | 0.0430 |
|  | Yes | Ref. |  |  | 1.000（Ref.） | Ref. |  |
| Self-rated economic status | Good | 0.485 | 0.626 | 0.599 | 1.623 | 0.476-5.536 | 0.439 |
|  | Fair | 0.770 | 0.347 | 4.927 | 2.160 | 1.094-4.264 | 0.026 |
|  | Poor | Ref. |  |  | 1.000（Ref.） | Ref. |  |
| Communication with children | >3 times/week | 0.002 | 0.564 | 0 | 1.002 | 0.331-3.026 | 0.998 |
|  | 1–3 times/ week | 0.676 | 0.413 | 2.684 | 1.967 | 0.876-4.417 | 0.101 |
|  | once a month | 0.578 | 0.291 | 3.936 | 1.783 | 1.007-3.157 | 0.047 |
|  | less than once per month | Ref. |  |  | 1.000（Ref.） | Ref. |  |
| Cognitive impairment | No | -0.812 | 0.333 | 5.954 | 0.444 | 0.231-0.852 | 0.015 |
|  | Yes | Ref. |  |  | 1.000（Ref.） | Ref. |  |
| Smoking | No | -0.643 | 0.321 | 4.010 | 0.526 | 0.28-0.986 | 0.045 |
|  | Yes | Ref. |  |  | 1.000（Ref.） | Ref. |  |
| Pain | No | -0.746 | 0.317 | 5.555 | 0.474 | 0.255-0.882 | 0.018 |
|  | Yes | Ref. |  |  | 1.000（Ref.） | Ref. |  |
| C4 vs. C2 |  |  |  |  |  |  |  |
| Perceived loneliness | No | -0.955 | 0.338 | 7.989 | 0.385 | 0.198-0.746 | 0.005 |
|  | Yes | Ref. |  |  | 1.000（Ref.） | Ref. |  |
| Smoking | No | -0.784 | 0.329 | 5.673 | 0.456 | 0.239-0.87 | 0.017 |
|  | Yes | Ref. |  |  | 1.000（Ref.） | Ref. |  |
| Depression | No | -0.747 | 0.333 | 5.033 | 0.474 | 0.247-0.91 | 0.025 |
|  | Yes | Ref. |  |  | 1.000（Ref.） | Ref. |  |
| C4 vs. C3 |  |  |  |  |  |  |  |
| Social Support Score | - | 0.058 | 0.025 | 5.369 | 1.060 | 1.009-1.114 | 0.021 |
| Self-rated economic status | Good | 0.416 | 0.697 | 0.356 | 1.516 | 0.386-5.945 | 0.551 |
|  | Fair | -0.827 | 0.407 | 4.119 | 0.438 | 0.197-0.972 | 0.042 |
|  | Poor | Ref. |  |  | 1.000（Ref.） | Ref. |  |
| Grandchild Caregiving | No | 0.706 | 0.344 | 4.211 | 2.027 | 1.032-3.979 | 0.040 |
|  | Yes | Ref. |  |  | 1.000（Ref.） | Ref. |  |
| Communication with children | >3 times/week | 0.472 | 0.611 | 0.597 | 1.604 | 0.484-5.314 | 0.440 |
|  | 1–3 times/ week | -0.399 | 0.494 | 0.653 | 0.671 | 0.255-1.766 | 0.419 |
|  | once a month | -0.901 | 0.395 | 5.191 | 0.406 | 0.187-0.882 | 0.023 |
|  | less than once per month | Ref. |  |  | 1.000（Ref.） | Ref. |  |
| Pain | No | 1.033 | 0.395 | 6.835 | 2.811 | 1.295-6.099 | 0.009 |
|  | Yes | Ref. |  |  | 1.000（Ref.） | Ref. |  |
| Depression | No | -0.755 | 0.383 | 3.883 | 0.470 | 0.222-0.996 | 0.049 |
|  | Yes | Ref. |  |  | 1.000（Ref.） | Ref. |  |

C1: low-level neglect; Class 2: selective mild neglect; Class 3: moderate neglect; Class 4: severe neglect.

**
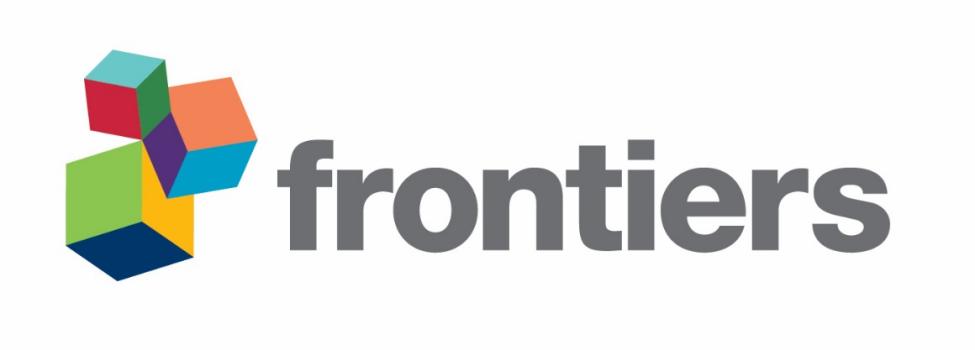
**
