# Supplementary material for: Latent profile analysis of self-neglect and associated factors among rural older adults with chronic diseases: a cross-sectional study
Source: Front Public Health. 2026 Jan 28;14:1738418. doi: 10.3389/fpubh.2026.1738418 (PMC12897509; doi:10.3389/fpubh.2026.1738418)
Supplement: Supplementary file 5 [file Table_5.docx]

Supplementary Material

**Supplementary Table 5. Interaction effects of internal vulnerabilities and external risks on latent profiles of ESN.**

| **Group comparison** | **Variables** | ***β*** | **Standard error** | **Wald *χ^2^* value** | | **OR** | **95%CI** | ***p* value** |
| --- | --- | --- | --- | --- | --- | --- | --- | --- |
| C2 vs. C1 | Living alone × Cognitive impairment | -0.76 | 0.622 | | 1.493 | 0.467 | 0.138-1.583 | 0.222 |
|  | Social support score × Depression | 0.048 | 0.034 | | 1.995 | 1.049 | 0.982-1.121 | 0.158 |
|  | Age × Pain | 0.061 | 0.030 | | 4.089 | 1.063 | 1.002-1.127 | **0.043** |
|  | Age × Social support score | -0.001 | 0.002 | | 0.133 | 0.999 | 0.995-1.003 | 0.716 |
|  | Age × Self-rated health | 0.003 | 0.023 | | 0.015 | 1.003 | 0.958-1.050 | 0.902 |
| C3 vs. C1 | Living alone × Cognitive impairment | 0.086 | 0.694 | | 0.015 | 1.090 | 0.279-4.249 | 0.902 |
|  | Social support score × Depression | 0.032 | 0.043 | | 0.557 | 1.033 | 0.949-1.123 | 0.456 |
|  | Age × Pain | 0.117 | 0.048 | | 5.798 | 1.124 | 1.022-1.236 | **0.016** |
|  | Age × Social support score | 0.003 | 0.003 | | 1.720 | 1.003 | 0.998-1.009 | 0.190 |
|  | Age × Self-rated health | 0.041 | 0.029 | | 2.018 | 1.042 | 0.984-1.103 | 0.155 |
| C4 vs. C1 | Living alone × Cognitive impairment | -0.329 | 0.799 | | 0.169 | 0.720 | 0.150-3.446 | 0.681 |
|  | Social support score × Depression | 0.039 | 0.040 | | 0.964 | 1.040 | 0.962-1.125 | 0.326 |
|  | Age × Pain | 0.083 | 0.045 | | 3.459 | 1.087 | 0.996-1.186 | 0.063 |
|  | Age × Social support score | 0.001 | 0.003 | | 0.056 | 1.001 | 0.995-1.006 | 0.814 |
|  | Age × Self-rated health | 0.031 | 0.030 | | 1.088 | 1.031 | 0.973-1.093 | 0.297 |
| C3 vs. C2 | Living alone × Cognitive impairment | 0.846 | 0.653 | | 1.682 | 2.331 | 0.649-8.375 | 0.195 |
|  | Social support score × Depression | -0.016 | 0.040 | | 0.155 | 0.984 | 0.91-1.065 | 0.694 |
|  | Age × Pain | 0.056 | 0.047 | | 1.383 | 1.057 | 0.963-1.161 | 0.240 |
|  | Age × Social support score | 0.004 | 0.002 | | 2.944 | 1.004 | 0.999-1.009 | 0.086 |
|  | Age × Self-rated health | 0.038 | 0.028 | | 1.884 | 1.039 | 0.984-1.098 | 0.170 |
| C4 vs. C2 | Living alone × Cognitive impairment | 0.432 | 0.759 | | 0.323 | 1.540 | 0.348-6.821 | 0.570 |
|  | Social support score × Depression | -0.009 | 0.037 | | 0.053 | 0.992 | 0.922-1.066 | 0.817 |
|  | Age × Pain | 0.022 | 0.044 | | 0.259 | 1.023 | 0.938-1.114 | 0.611 |
|  | Age × Social support score | 0.001 | 0.003 | | 0.293 | 1.001 | 0.996-1.007 | 0.588 |
|  | Age × Self-rated health | 0.028 | 0.029 | | 0.951 | 1.029 | 0.972-1.088 | 0.330 |
| C4 vs. C3 | Living alone × Cognitive impairment | -0.415 | 0.813 | | 0.260 | 0.661 | 0.134-3.248 | 0.610 |
|  | Social support score × Depression | 0.007 | 0.045 | | 0.026 | 1.007 | 0.923-1.099 | 0.871 |
|  | Age × Pain | -0.034 | 0.056 | | 0.357 | 0.967 | 0.866-1.079 | 0.550 |
|  | Age × Social support score | -0.003 | 0.003 | | 0.889 | 0.997 | 0.991-1.003 | 0.346 |
|  | Age × Self-rated health | -0.010 | 0.032 | | 0.103 | 0.990 | 0.929-1.054 | 0.748 |

C1: low-level neglect; Class 2: selective mild neglect; Class 3: moderate neglect; Class 4: severe neglect.

**
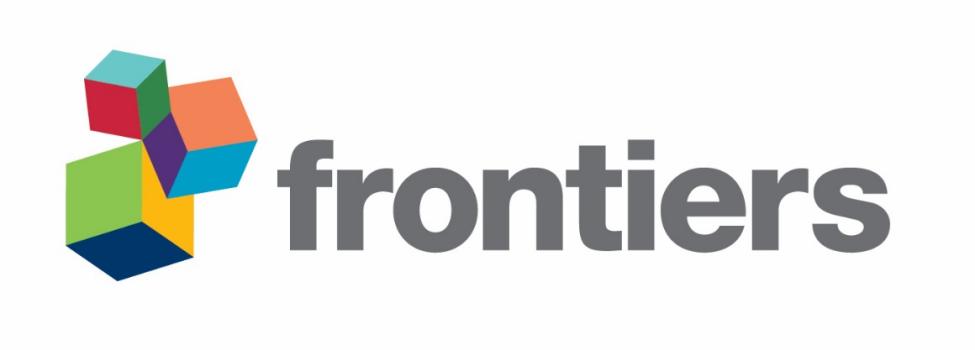
**
